# MARVELD1 Inhibits Nonsense-Mediated RNA Decay by Repressing Serine Phosphorylation of UPF1

**DOI:** 10.1371/journal.pone.0068291

**Published:** 2013-06-27

**Authors:** Jianran Hu, Yu Li, Ping Li

**Affiliations:** 1 School of Life Science and Technology, Harbin Institute of Technology, Harbin, China; 2 School of Municipal and Environmental Engineering, Harbin Institute of Technology, Harbin, China; German Cancer Research Center, Germany

## Abstract

We have observed low expression levels of MARVELD1, a novel tumor repressor, in multiple tumors; however, its function in normal cells has not been explored. We recently reported that MARVELD1 interacts with importin β1, which plays an important role in nonsense-mediated RNA decay(NMD). Here, we demonstrate that MARVELD1 substantially inhibits nonsense-mediated RNA decay by decreasing the pioneer round of translation but not steady-state translation, and we identify MARVELD1 as an important component of the molecular machinery containing UPF1 and Y14. Furthermore, we determined the specific regions of MARVELD1 and UPF1 responsible for their interaction. We also showed that MARVELD1 promotes the dissociation of SMG1 from UPF1, resulting in the repression of serine phosphorylation of UPF1, and subsequently blocks the recruitment of SMG5, which is required for ensuing SMG5-mediated exonucleolytic decay. Our observations provide molecular insight into the potential function of MARVELD1 in nonsense-mediated RNA decay.

## Introduction

Nonsense-mediated mRNA decay (NMD) is a quality-control mechanism to eliminate premature termination codon (PTC)-containing mRNAs that synthesize truncated proteins [1]. As many as 30% of all mutated protein-coding mRNAs are degraded by NMD [2]. During the pioneer round of translation, newly synthesized mRNAs bound at the 5’ caps by the cap-binding protein complex (CBC) CBP80-CBP20 are marked by exon junction complexes (EJCs) at exon–exon junctions [3]. Before this occurs, the transient and/or weak interaction between cap-bound CBP80 and UPF1, a key NMD factor, promotes NMD if the mRNA contains a premature termination codon (PTC) 50-55 nucleotides upstream of an EJC-bound exon–exon junction [4]. The phosphorylation of UPF1 by suppressor of morphogenetic effect on genitalia 1 (SMG1), a phosphatidylinositol kinase (PIK)-related serine/threonine protein kinase, triggers additional rounds of translation initiation repression [5]. SMG-6 binds to phosphorylated threonine 28 at the N-terminus of UPF1, and the SMG5-SMG7 or SMG5-PNRC2 complex binds to phosphorylated serine 1096 at the C-terminus of UPF1. The interactions between the SMG5 complexes and UPF1 result in sequential remodeling of the mRNA surveillance complex for NMD induction and recycling of the ribosome, release factors and NMD factors [6]. Downregulating the expression of UPF1 using siRNA upregulates the levels of many NMD target transcripts in HeLa cells [7]. Although many NMD factors are known, very few modulators of UPF1 have been characterized.

MARVELD1 (MARVEL domain containing 1) is a novel MARVEL domain-containing protein. We previously reported that MARVELD1 localized to interphase cell nuclei and was downregulated in multiple tumor tissues [8]. The overexpression of MARVELD1 inhibited proliferation and enhanced chemosensitivity in hepatocellular carcinoma cells [9]. We also identified the interaction between MARVELD1 and importin β1 by liquid chromatography-tandem mass spectrometry analysis and coimmunoprecipitation in HeLa cells [10]. Importin β has been suggested to regulate the RNA cap-binding activity of CBC by binding to the CBP20 subunit and affecting the remodeling of the pioneer translation initiation complex [11,12]. CBC-bound but not eIF4E-bound mRNAs are the potential targets of NMD [1]. Thus, we infer that MARVELD1 may participate in the biological functions of importin β1 and CBC. Hereby, we hypothesized that there may be a relationship between MARVELD1 and NMD.

In this study, to investigate the functional effect of MARVELD1 on NMD, we determined the expression of reporter genes and the target transcripts of NMD when MARVELD1 was overexpressed or depleted. We observed that MARVELD1 clearly inhibited NMD. Furthermore, we validated that MARVELD1 inhibits pioneer round translation but not steady-state translation. To elucidate the molecular mechanism, we analyzed the relationship of MARVELD1 with NMD factors, such as SMG1, UPF1, CBP80, SMG5 and Y14. We lastly found that the C-terminus of UPF1 was immunoprecipitated by anti-MARVELD1. Furthermore, overexpression of MARVELD1 blocked the serine phosphorylation of UPF1 and prevented the interaction between UPF1 and SMG5, whereas knockdown of MARVELD1 enhanced both. These findings indicate that MARVELD1 regulates NMD by inhibiting the interaction between UPF1 and SMG5.

## Materials and Methods

### Plasmid constructs and siRNA

The NMD reporter plasmids (pmCMV-G1 Norm/Ter, pmCMV-GPx1 Norm/Ter and phCMV-mup) and the plasmids expressing Flag-UPF1 and Flag-Y14 were kindly provided by Lynne E. Maquat [13] and Yasumasa Ishida [14], respectively. To construct MARVELD1(1-33), MARVELD1(1-69), MARVELD1(1-100), MARVELD1(1-144) and MARVELD1(Δ34-69), the plasmid pcDNA3.1-MARVELD1-V5 [8] was amplified using the primers in the Table S1, and then the PCR products were ligated into pcDNA3.1-Flag vector. The expression plasmids for UPF1(242-1118), UPF1(242-797) and UPF1(788-1118) were constructed in the pCMV-Flag vectors using the primers in the Table S1.

To deplete the expression of MARVELD1 and UPF1, the siRNAs were used as follows: siMARVELD1 5’-r(AUU GGA ACC AGG CUU CUG G)-3’ [9], siMARVELD1-2 5’-r(CCU CAA GGA UUA CCC GCU C)-3’, siUPF1-1 5’-r(GAU GCA GUU CCG CUC CAU U)-3’ [15] and siUPF1-2 5’-r(GAG AAU CGC CUA CUU CAC U)-3’ [16].

### Cell culture and transfection

All cell lines used in this study were obtained from the American Type Culture Collection. HEK293, HeLa and HepG2 cells grown in DMEM supplemented with 10%(v/v) fetal calf serum and 2 mM L-glutamine. A549 and H520 cells were cultured in RPM1640 medium supplemented with 10% fetal calf serum and 2 mM L-glutamine. Cells were transiently transfected with vitro-synthesized siRNA or indicated plasmids using Lipofectamine 2000 according to manufacturer’s instructions (Invitrogen). To obtain cell lines stably overexpressing MARVELD1, A549, HEK293 and HepG2 cells transfected with pcDNA3.1-MARVELD1-V5 were screened in complete medium with 500 μg/ml G418 (Invitrogen) for 14 days, and positive clones were selected and identified by western blot.

### RNA stabilization assay and real-time PCR

To assess RNA stabilization, the RNA polymerase II inhibitor 5,6-dichlorobenzimidazole-1-β-D-ribofuranoside (DRB, sigma) was added at 100 μg/ml to suppress transcription. Total RNA was then serially collected at the indicated time points, and cDNA was synthesized using the High Capacity cDNA Revers Transcription Kit (Applied Biosystems). The real-time PCR was performed using the SYBR@Premix Ex Taq™ (Perfect Real Time) (Takara) by the Applied Biosystems 7500 Real-Time PCR system. PCR conditions were: 95°C 1 min, 95°C 10 s, 60°C 34 s for 40 cycles. The primers were listed in Table S2.

### Immunoprecipitation

Immunoprecipitations were performed as described previously [17] with slight modification. Briefly, approximately 1×10^7^ cells were lysed in 700 μl IP buffer (50 mM Tris–HCl pH 8.0, 150 mM NaCl, 1% Triton X-100, 1 mM EDTA, 1 mM DTT) containing 10 μg/ml aprotinin, 10 μg/ml leupeptin, 1 mM PMSF and 1% phosphatase inhibitor cocktail (Sigma) on ice for 30 min. Immunoprecipitations were performed in the presence or absence of RNase A (Fermentas). The lysate was centrifuged at 13,000 g for 15 min at 4°C followed by determination of protein concentration. Ten percent of the supernatant was kept as input, and the remaining lysate was rotated with 10 μg primary antibody or normal rabbit/mouse IgG overnight at 4°C before being mixed with 20 μl of protein A beads. The sample was then rotated at 4°C for another 3 hours. The precipitated proteins were eluted with sample buffer after being washed 5 times with IP buffer and then separated by SDS-PAGE. The following antibodies were used: Flag (Sigma), V5 (Sigma), UPF1 (Abcam), SMG1 (Abcam), SMG5 (Abcam), MARVELD1 (Abcam), CBP80 (Santa Cruz), CBP20 (Santa Cruz), importin β1 (Santa Cruz), phosphoserine (BD), GAPDH (Santa Cruz), Y14 (Abcam), ATF-4 (Boster), ATF-3 (Bioss), Pim3 (Bioss), arhgef18 (Abcam), and Frs2 (Abcam).

## Results

### MARVELD1 inhibits NMD

**Figure 1:**
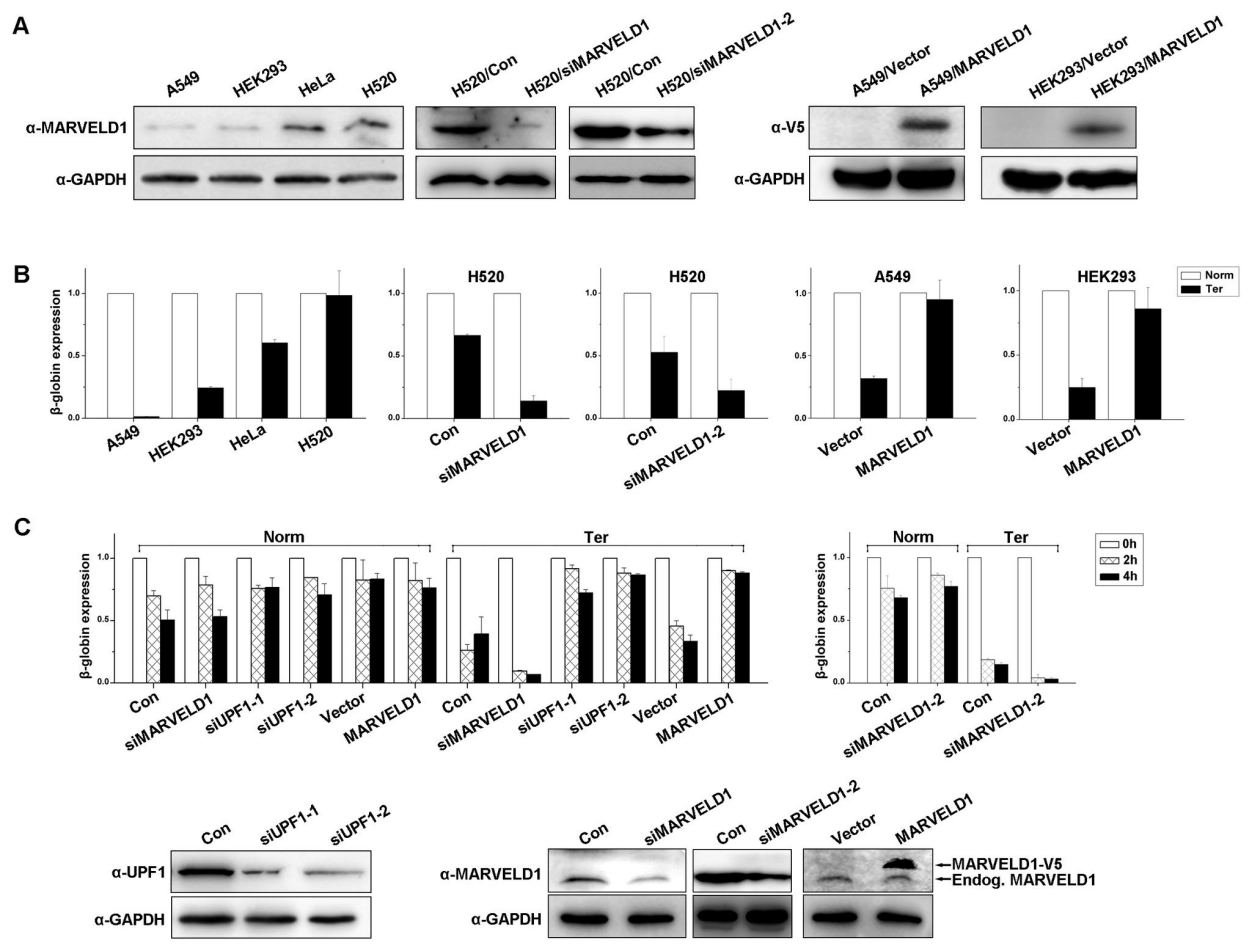
MARVELD1 is required for the stabilization of the NMD reporter transcript. (A) Expression analysis of MARVELD1 by western blotting. H520 cells were transiently transfected with two independent siRNAs against *MARVELD1* or a negative control siRNA. A549 and HEK293 cells were stably transfected with pcDNA3.1-MARVELD1-V5/His (HEK293/MARVELD1) or the control plasmid pcDNA3.1-V5/His (HEK293/Vector). (B) A549, HEK293, HeLa and H520 cells as well as MARVELD1-depleted H520 cells, A549/MARVELD1 cells and HEK293/MARVELD1 cells were transiently cotransfected with either pmCMV-Gl Norm or pmCMV-Gl Ter and phCMV-MUP. The mRNA level of β-globin was assessed by real-time PCR of cDNA. The β-globin mRNA was normalized to the expression of Mup mRNA, and the normalized level of G1 (β-globin) Norm mRNA was defined as 1.0. (C) HeLa cells with depleted MARVELD1, overexpressed MARVELD1 or transiently depleted UPF1 were cotransfected with either pmCMV-Gl Norm or pmCMV-Gl Ter and phCMV-MUP, and the level of β-globin mRNA was assessed by treating cells with DRB and serially assessing globin mRNA levels by real-time PCR of cDNA. The β-globin mRNA was normalized to the expression of Mup mRNA, and the normalized level of G1 (β-globin) Norm and Ter mRNA at 0 h was defined as 1.0. The effects of siRNA-mediated knockdown of UPF1 and MARVELD1 were determined by western blotting, and GAPDH was used as an internal control. All of the experiments were performed with three replicates. The average ± S.E. (*error bars*) is displayed in column diagrams of (B) and (C).

To investigate the effect of MARVELD1 on NMD activity, we stably overexpressed MARVELD1-V5 in A549 and HEK293 cells and depleted MARVELD1 expression in H520 and HeLa cells using specific siRNAs (Figure 1A and 1C). To assess NMD activity, these cells were transiently cotransfected with the phCMV-MUP reference plasmid and the reporter β-globin construct containing a premature termination codon at position 39 (pmCMV-G1 Ter) or the wild-type construct as a control (pmCMV-G1 Norm) [13]. Total RNA was collected 48 hours after transfection, and the expression level of β-globin mRNA was detected using real-time PCR of cDNA (Figure 1B). The β-globin mRNA level was normalized to the expression of Mup mRNA, and the normalized level of G1 (β-globin) Norm mRNA was defined as 1.0. The level of mutated β-globin transcripts (Ter) was very low in A549 and HEK293 cells and substantially increased in HeLa and NCI-H520 cells. The overexpression of MARVELD1 in A549 and HEK293 cells greatly increased the level of mutated β-globin transcripts (Ter), whereas knockdown of MARVELD1 in HeLa and H520 cells reduced the expression of the mutated β-globin transcripts (Ter) (Figure 1B and Figure 1C). Similar results were obtained in HepG2 cells and by transfection with a second NMD reporter construct, pmCMV-GPx1, which contains a premature termination codon at position 46 [13] (Figure S1). Furthermore, HeLa cells depleted of MARVELD1, with overexpressed MARVELD1, or depleted of UPF1 were all cotransfected with either pmCMV-G1 Norm or pmCMV-G1 Ter and phCMV-Mup. NMD activity was then assessed by inhibiting transcription with DRB (an RNA II polymerase inhibitor) and serially determining globin transcript expression by real-time PCR. The level of β-globin mRNA was first normalized to the expression of Mup mRNA, and then the normalized level of G1 (β-globin) Norm or Ter mRNA at 0 h was defined as 1.0. As Figure 1C shows, the stabilization of mutated β-globin transcripts (Ter) was remarkably increased in HeLa cells overexpressing MARVELD1 compared with the cells transfected with control plasmid or siRNAs and was similar to the stabilization in the cells with a low expression of UPF1. The depletion of MARVELD1 greatly reduced the stabilization of mutated β-globin transcripts (Ter) compared with the stabilization in HeLa cells transfected with control siRNA.

**Figure 2:**
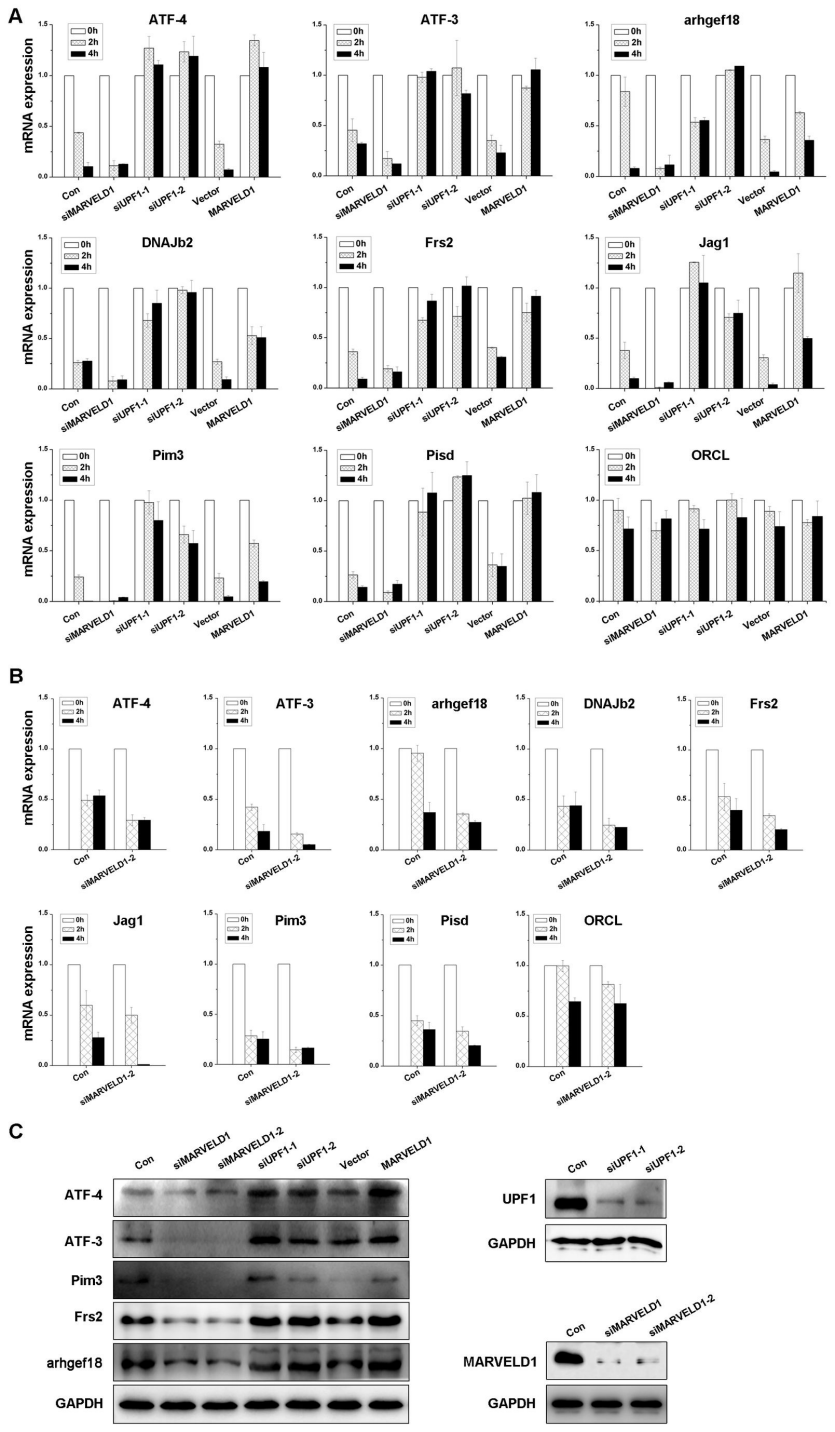
MARVELD1 stabilizes NMD targets. (A) The stabilization of eight NMD targets and a control transcript were assessed in HeLa cells with depleted or overexpressed MARVELD1 or transiently depleted UPF1. (B) The stabilization of the transcripts was analyzed in HeLa cells transfected with a second independent siRNA against MARVELD1 using real-time PCR. Total RNA was collected in the presence of DRB at 0, 2, and 4 h, and real-time PCR was performed to assess the mRNA level. The results were normalized to GAPDH RNA expression, and the normalized mRNA level at 0 h was defined as 1.0. Average ± S.E. (*error bars*) of duplicate experiments are displayed. (C) The expression of five NMD target genes was analyzed by western blotting when MARVELD1 or UPF1 was depleted or MARVELD1 was overexpressed. The effects of siRNA-mediated knockdown of UPF1 and MARVELD1 were also determined. GAPDH was used as an internal control.

To further confirm the effect of MARVELD1 on NMD, we examined the stabilization of eight endogenous NMD targets (ATF4, arhgef18, ATF3, DNAJb2, Pim3, Jag1, Frs2 and Pisd) and a control mRNA (ORCL) not degraded by NMD [18,19] in the presence of the transcription inhibitor DRB. The levels of these transcripts were normalized to GAPDH RNA expression, and the normalized mRNA level at 0 h was defined as 1.0. These NMD targets were all upregulated in HeLa cells with MARVELD1 overexpression or UPF1 depletion and downregulated in the cells with MARVELD1 depleted (Figure 2A). However, alteration of MARVELD1 expression did not affect the level of the control transcript ORCL. Additionally, the expression of five target genes was analyzed by western blotting, and the results were consistent with the observations by real-time PCR (Figure 2B). The results indicated that MARVELD1 represses NMD activity.

The pioneer round of translation is distinct from steady-state translation. During the pioneer round of translation, the 5’-cap of mRNA binds to the CBP80-CBP20 complex (CBC), and one or more exon-junction complexes (EJCs) are detectable on the spliced CBC-bound mRNAs [3]. By the time the CBC is replaced by eIF4E and EJCs are no longer detectable, steady-state translation is initiated. The pioneer round of translation supports NMD [3], and quantitation of the levels of G1 and Mup mRNA by RT-PCR is used as a measure of the pioneer round of translation [20]. The above real-time PCR results indicated that MARVELD1 represses the pioneer round of translation. To identify whether MARVELD1 also inhibits steady-state translation, luciferase activity assays were performed (Figure S2) as reported by Chiu [20]. The results showed that MARVELD1 does not affect steady-state translation.

### MARVELD1 interacts with proteins involved in NMD

**Figure 3:**
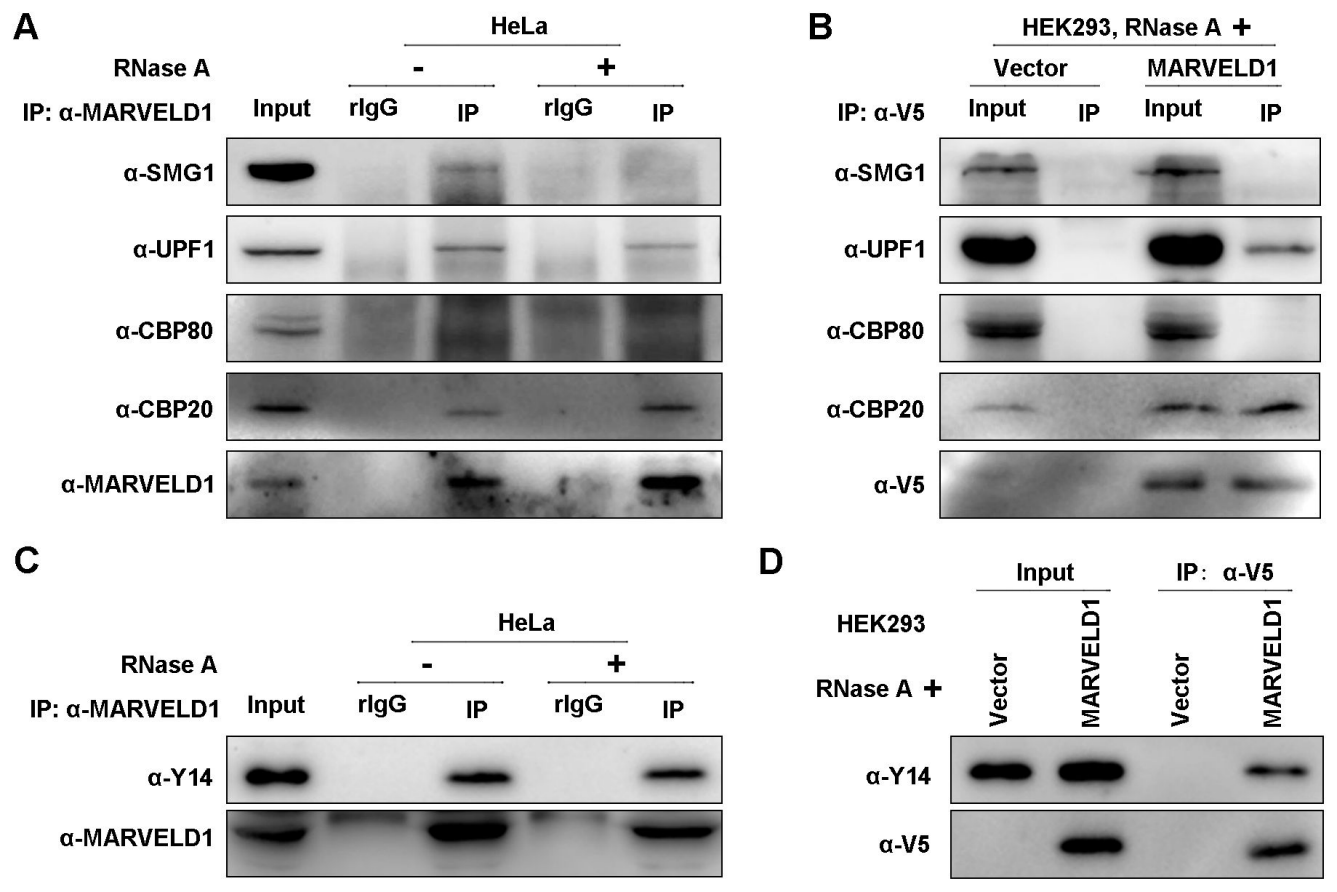
MARVELD1 interacts with proteins involved in NMD. (A) Whole cell extract of HeLa cells treated with or without RNase A was immunoprecipitated by anti-MARVELD1 or, as a control for nonspecific IP, rabbit (r) IgG. (B) HEK293/MARVELD1 cells or the negative control cells HEK293/Vector were immunoprecipitated by anti-V5 in the presence of RNase A. (C) Extracts of HeLa cells were immunoprecipitated by anti-MARVELD1 or, as a control for nonspecific IP, rabbit (r) IgG in the presence or absence of RNase A. (D) Lysates of HEK293/MARVELD1 cells or the negative control cells HEK293/Vector were immunoprecipitated by anti-V5 in the presence of RNase A. All of the experiments were performed independently at least twice.

Because MARVELD1 inhibited NMD activity, we aimed to determine the relationship between MARVELD1 and NMD factors. Using extracts of HeLa cells with high endogenous expression of MARVELD1, UPF1 and CBP20 were immunopurified by anti-MARVELD1 but not rabbit IgG (rIgG) (Figure 3A), which was used as a control for nonspecific immunopurification. CBP80 did not purify with MARVELD1. RNase A degrades cellular RNA and was used to ensure that the interaction was stable in the absence of RNA, and the effect of RNase A is shown in Figure S3. SMG1 was immunopurified by anti-MARVELD1 only in the presence of RNA. Meanwhile, lysates of HEK293 cells stably transfected with MARVELD1-V5 or control plasmid were immunoprecipitated using anti-V5 (Figure 3B). Western blotting confirmed that exogenous MARVELD1 also interacted with UPF1 and CBP20, but not SMG1 and CBP80, in the presence of RNase A. Thus, MARVELD1 may not be a component of a complex containing SMG1 and CBP80, such as SURF, which primarily consists of SMG1, UPF1, eRF1 and eRF3 [1], but appeared to be a part of a complex containing UPF1 and/or CBP20.

Based on these observations, we next investigated whether MARVELD1 participates in the function of another important NMD complex, the EJC. Y14, an EJC component, is a RNA-binding protein and forms a stable heterodimer with Mago [21,22,23]. The Y14-Mago heterodimer is part of the EJC core and recruits several key factors of NMD [21,22,23,24]. In the presence of RNase A, coimmunoprecipitations were performed in HeLa cells or HEK293 cells stably overexpressing MARVELD1-V5. As Figure 3C and D show, both endogenous and exogenous MARVELD1 was immunoprecipitated with endogenous Y14, whereas coimmunoprecipitation was not observed in the control cells. Additionally, exogenous Flag-Y14 was immunoprecipitated by both endogenous and exogenous MARVELD1 in the presence of RNase A (Figure S4). Thus, MARVELD1 likely coregulates NMD with the EJC.

### Amino acids 34-69 are necessary for MARVELD1 to interact with UPF1

**Figure 4:**
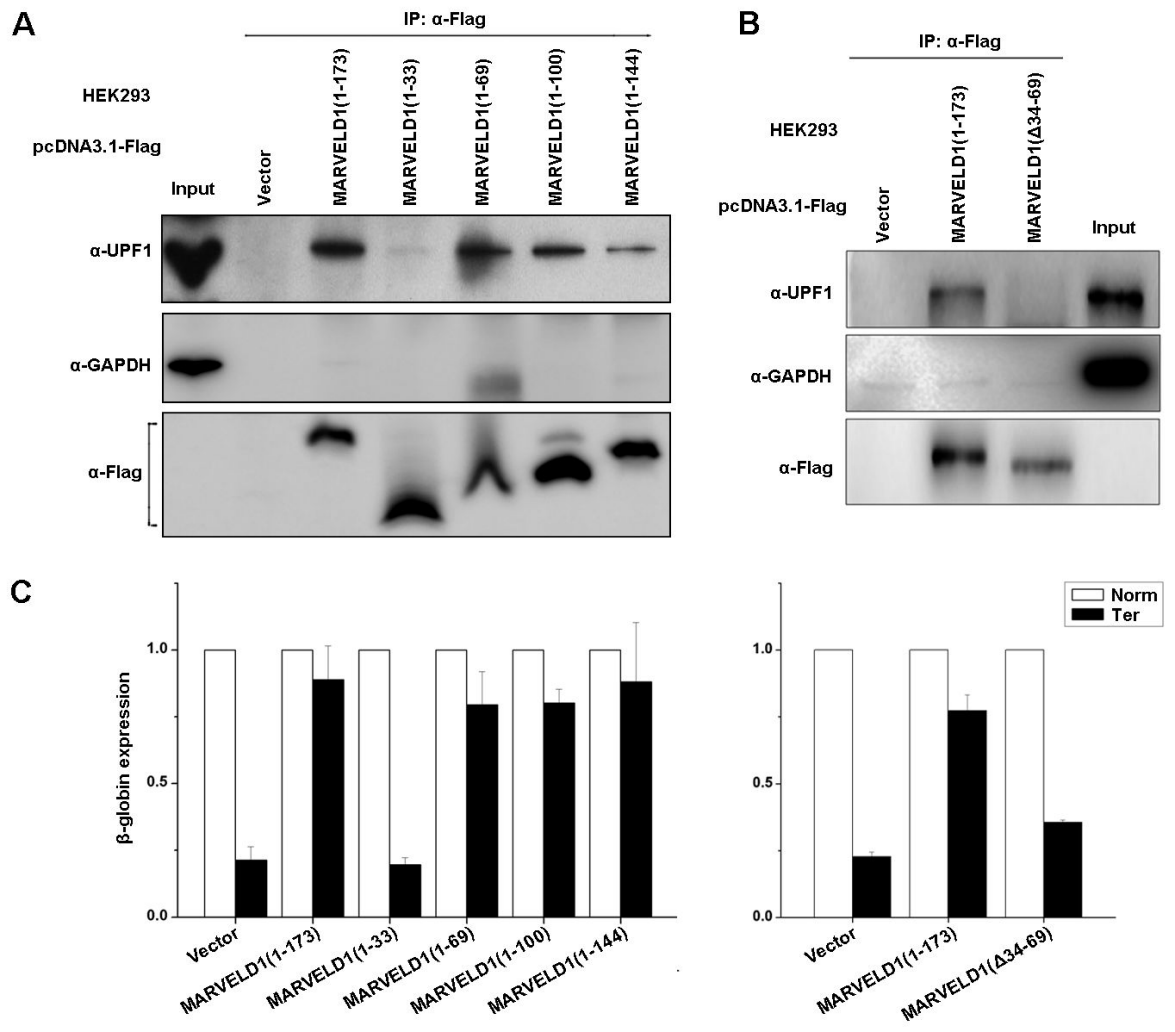
Amino acids 34-69 of MARVELD1 are required to interact with UPF1. (A) Lysates of HEK293 cells that had been transiently transfected with the specified pcDNA3.1-Flag-MARVELD1 construct or the control plasmid pcDNA3.1-Flag were analyzed by western blotting using the specified antibody before or after IP with anti-Flag. (B) Lysates of HEK293 cells that had been transiently transfected with pcDNA3.1-Flag-MARVELD1(1-173), pcDNA3.1-Flag-MARVELD1(Δ34-69) or the control plasmid pcDNA3.1-Flag (Vector) were immunoprecipitated by anti-Flag. Western blotting was performed using the specified antibody before or after IP. (C) HEK293 cells that were transiently cotransfected with the specified pCMV-Flag-MARVELD1 plasmid, either pmCMV-Gl Norm or pmCMV-Gl Ter and phCMV-MUP. Real-time PCR was used to normalize the level of G1 (β-globin) mRNA relative to Mup mRNA, and the normalized level of G1 (β-globin) Norm mRNA in the presence of each pcDNA3.1-Flag plasmid was defined as 1.0. The average ± S.E. (*error bars*) is displayed. All of the experiments were performed at least twice.

Because MARVELD1 binds to UPF1 and Y14 and represses NMD activity and because UPF1 is an important protein responsible for the initiation of NMD, it was necessary to determine the specific region of MARVELD1 that is required for its interaction with UPF1. To define the domain, we generated four deletion variants of N-terminally Flag-tagged full-length MARVELD1, which consists of amino acids 1-173. These variants were MARVELD1(1-33), MARVELD1(1-69), MARVELD1(1-100) and MARVELD1(1-144). Lysates of HEK293 cells transiently transfected with one of the variants or the control plasmid pCMV-Flag were immunoprecipitated under conditions where the Flag-MARVELD1 proteins were expressed at similar levels (Figure 4A, α-Flag). The results indicated that UPF1 was immunopurified with Flag-MARVELD1(1-173), Flag-MARVELD1(1-69), and Flag-MARVELD1(1-144), but not Flag-MARVELD1(1-33) or the negative-control protein GAPDH (Figure 4A).

To further determine the effect of the MARVELD1 deletion variants on NMD, HEK293 cells transiently expressing these deletion proteins were cotransfected with the pmCMV-G1 test plasmid (Norm or Ter) and the phCMV-MUP reference plasmid. Total RNA was collected after 48 hours. Real-time PCR revealed that Flag-MARVELD1(1-173), Flag-MARVELD1(1-69) and Flag-MARVELD1(1-144), unlike Flag-MARVELD1(1-33), indeed repressed NMD: the level of β-globin mRNA containing a PTC at position 39 (Ter) was 80%-90% of normal in the presence of Flag-MARVELD1(1-173), Flag-MARVELD1(1-69) or Flag-MARVELD1(1-144) but less than 25% of normal in the presence of Flag-MARVELD(1-33) or Flag (Figure 4C, left panel). Therefore, the presence of amino acids 34-69 of MARVELD1 increased the level of PTC-containing β-globin mRNA more than 3-fold compared with the level when this region was absent.

The above results implied that the region containing amino acids 34 to 69 was important for the inhibitory effect of MARVELD1 on NMD. We constructed another deletion variant, Flag-MARVELD1(Δ34-69), which was deleted for amino acids 34 to 69. Coimmunoprecipitations were performed using lysates of HEK293 cells transiently transfected with pcDNA3.1-Flag-MARVELD1(1-173), pcDNA3.1-Flag-MARVELD1(Δ34-69) or the control plasmid pcDNA3.1-Flag. Western blotting indicated that MARVELD1 no longer associated with UPF1 when amino acids 34 to 69 were absent (Figure 4B). Additionally, real-time PCR results of the NMD reporter genes indicated that the level of PTC-containing β-globin mRNA was only approximately 30% of normal without amino acids 34 to 69 and was similar to the level in the presence of the control plasmid (Figure 4C, the right panel). That is, MARVELD1 did not inhibit NMD without amino acids 34 to 69. Therefore, amino acids 34 to 69 indeed contain the specific site required for the interaction between MARVELD1 and UPF1 and are responsible for the inhibitory effect of MARVELD1 on NMD.

### The C-terminus of UPF1 is required for the interaction with MARVELD1

**Figure 5:**
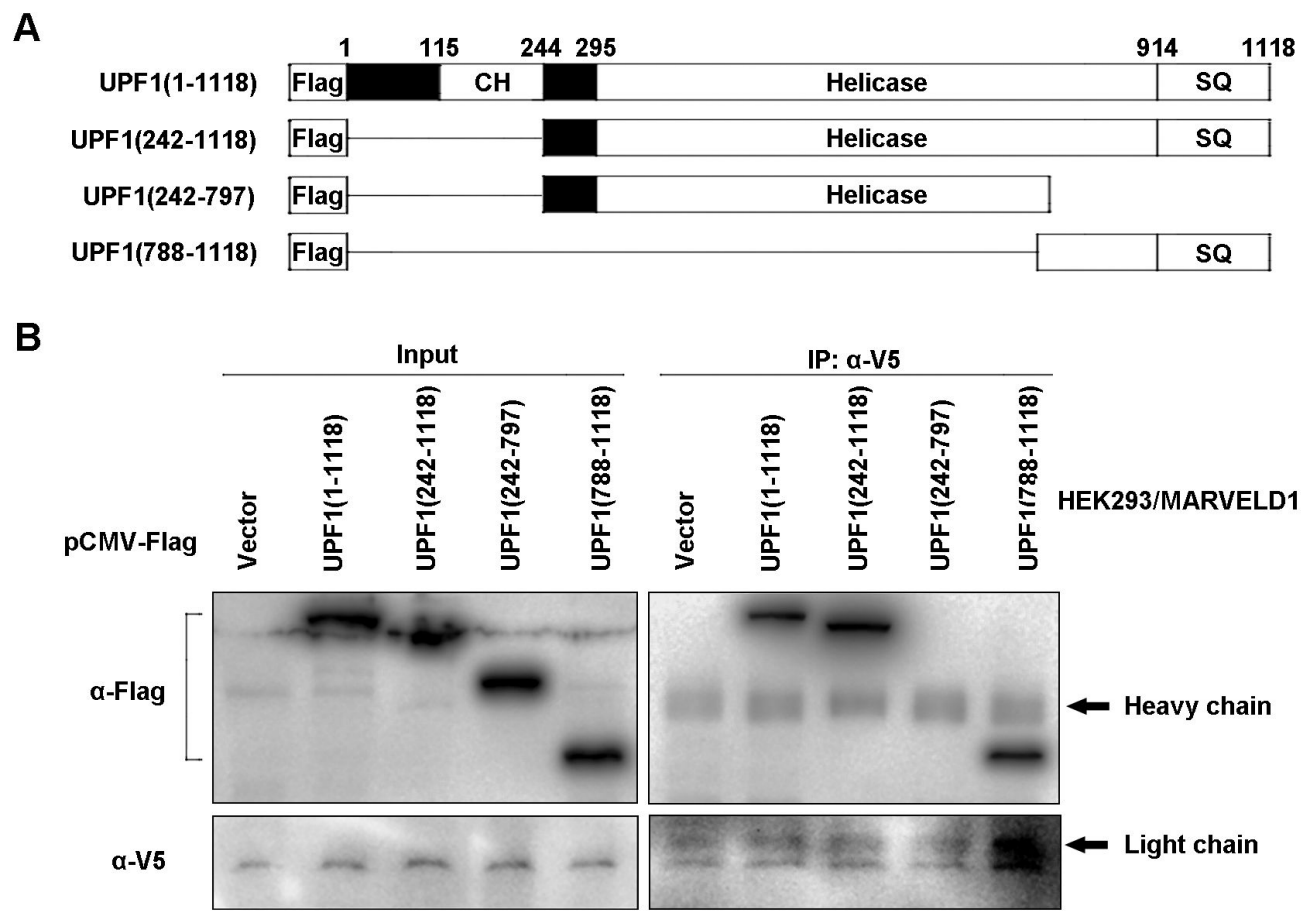
The C-terminal region of UPF1 is necessary to interact with MARVELD1. (A) Schematic representation of full-length Flag-UPF1(1–1118) and three Flag-UPF1 deletion variants. (B) HEK293/MARVELD1 cells were transiently transfected with different Flag-UPF1 deletion variants or the control plasmid pCMV-Flag and analyzed by coimmunoprecipitation using anti-V5. The results are representative of three independently performed experiments.

UPF1 is a key factor of NMD and contains several domains that are responsible for binding to several NMD-related molecules. For example, the cysteine-histidine-rich (CH) domain interacts with UPF2 and eRF3 [21], and the SQ-rich domain is indispensable for UPF1 to bind to the SMG5-SMG7 and SMG5-PNRC2 complexes [6]. To determine the mechanism by which MARVELD1 represses NMD, it was necessary to identify the specific region in the UPF1 protein that is required for the inhibitory effect of MARVELD1. Thus, three deletion variants of FLAG-UPF1 were generated (Figure 5A). These recombinant plasmids were transfected into HEK293 cells stably overexpressing MARVELD1-V5. Coimmunoprecipitations using anti-V5 indicated that MARVELD1 interacted with the deletion variants containing the C-terminal region of UPF1, including Flag-UPF1(1-1118), Flag-UPF1(242-1118) and Flag-UPF1(788-1118), but not Flag-UPF1(242-797), which did not contain the C-terminal region (Figure 5B). Hereby, the C-terminal region of UPF1 is necessary for its interaction with MARVELD1.

### MARVELD1 prevents UPF1 binding to SMG1

**Figure 6:**
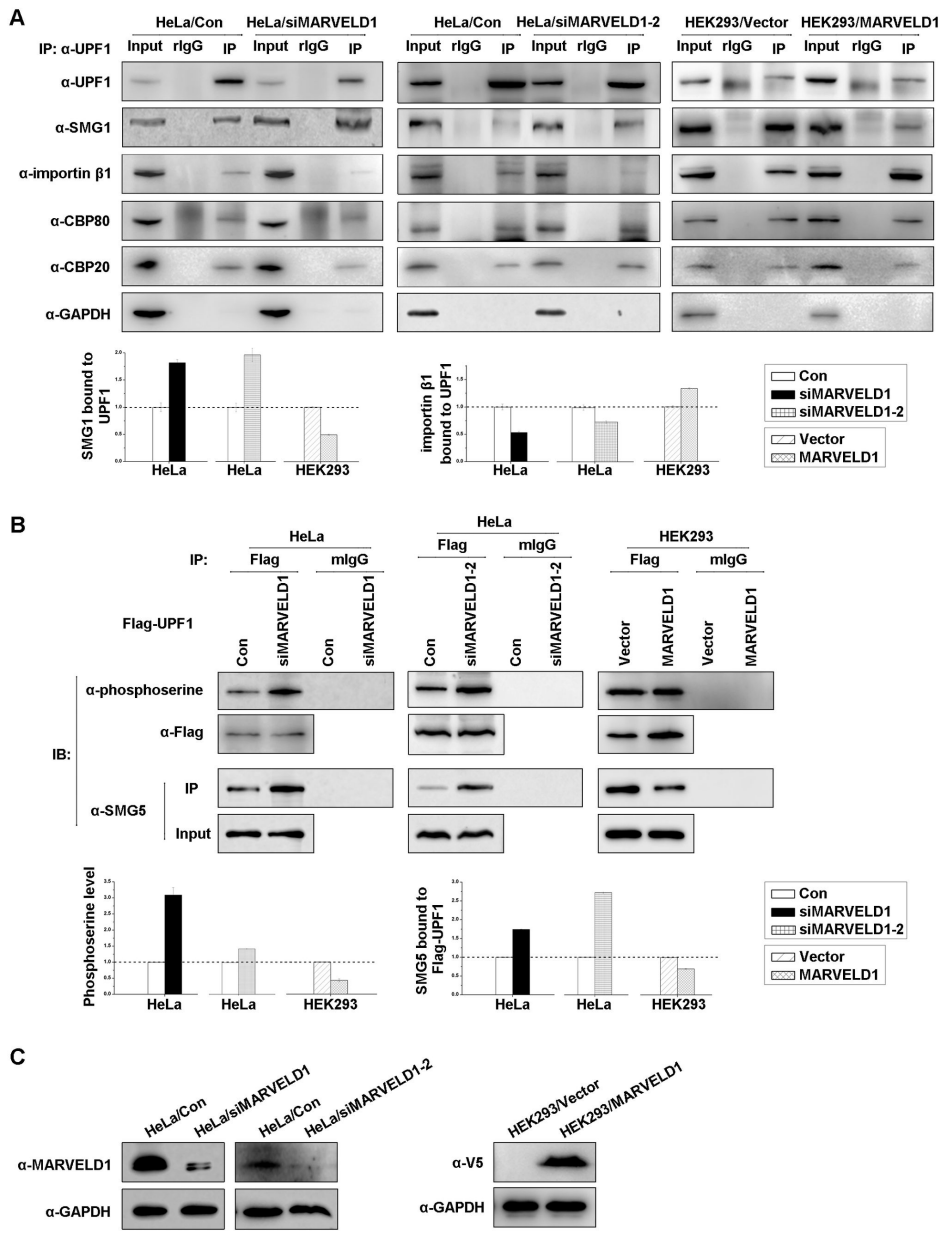
MARVELD1 represses the recruitment of SMG5 by inhibiting serine phosphorylation of UPF1. (A) HeLa cells transiently transfected with two independent siRNAs against *MARVELD1* or the negative control siRNA and HEK293/MARVELD1 or the control cells HEK293/Vector were analyzed by coimmunoprecipitation using anti-UPF1 or rabbit (r) IgG in the presence of RNase A. The analysis of the intensity of SMG1 and importin β1 immunoprecipitated by anti-UPF1 is displayed as histograms on the right. The coIPs of SMG1 and importin β1 were both normalized to the amount of UPF1 immunoprecipitated by anti-UPF1. (B) Lysates of HeLa cells transiently depleted of MARVELD1 and HEK293/MARVELD1 were transiently transfected with Flag-UPF1 and immunoprecipitated by anti-Flag or mouse (m) IgG. Western blotting was performed using anti-phosphoserine or anti-SMG5. The intensities of phosphoserine level and the SMG5 immunoprecipitated by anti-Flag were normalized to the total Flag-UPF1 and are displayed as histograms on the right. (C) The effects of siRNA-mediated knockdown of MARVELD1 and the overexpression of MARVELD1-V5 were assessed by western blotting. GAPDH was used as an internal control. The average ± S.E. (*error bars*) is displayed.

Because MARVELD1 inhibited NMD and interacted with UPF1, knockdown or overexpression of MARVELD1 was expected to affect the formation of UPF1-containing complexes involved in NMD. To test this prediction, lysates of HeLa cells transiently transfected with two siRNAs against *MARVELD1* mRNA or a negative control siRNA were immunoprecipitated using anti-UPF1 or, as a control for nonspecific IP, rabbit IgG (rIgG) (Figure 6A). As expected, depletion of MARVELD1 increased the coimmunoprecipitation of SMG1 by 1.7 and 1.9-fold and decreased the coimmunoprecipitation of importin β1 to 50% and 70% compared with the level observed in the control cells (Figure 6A). Moreover, as expected, overexpression of MARVELD1 in HEK293 cells reduced the amount of SMG1 bound to UPF1 to approximately 50% and enhanced the interaction of importin β1 and UPF1 by 1.3-fold compared with the level observed in the control cells (Figure 6A). However, the alteration of MARVELD1 expression did not disrupt the interaction of UPF1 and CBC.

### MARVELD1 blocks the serine phosphorylation of UPF1 and the recruitment of SMG5

As MARVELD1 inhibited the interaction of UPF1 and SMG1 and because the phosphorylation of UPF1 by SMG1 is an important event to trigger NMD [5,21], we hypothesized that the overexpression of MARVELD1 would decrease the phosphorylation level of UPF1 and subsequently reduce the recruitment of SMG5 and/or SMG6 [6], which associate with phospho-S1096 and -T28 of UPF1, respectively. In contrast, knockdown of MARVELD1 was expected to facilitate the phosphorylation of UPF1 and the binding of SMG5 and/or SMG6. To test this hypothesis, HeLa cells with MARVELD1 depleted and HEK293 cells stably overexpressing MARVELD1 were transiently transfected with pCMV-Flag-UPF1, and immunoprecipitations were performed using anti-Flag or, as a control for nonspecific immunoprecipitation, mouse IgG (mIgG). As negative controls, HeLa cells transfected with nonsense siRNA or HEK293 cells with control plasmid pcDNA3.1/V5-His were handled the same way. As Figure 6B shows, depletion of MARVELD1 indeed increased the serine phosphorylation level of Flag-UPF1 by approximately 3 and 1.5-fold, whereas overexpression of MARVELD1 reduced serine phosphorylation to approximately 50% of the level observed in the control cells (Figure 6B) after being normalized to the level of UPF1 in the whole cell extract. Immunoprecipitations were also performed to assess alterations in serine phosphorylation of endogenous UPF1 using anti-UPF1, and similar results were observed (Figure S5). However, the alterations in MARVELD1 expression did not affect the threonine phosphorylation status (data not shown). We therefore assessed the effect of MARVELD1 on the interaction of SMG5 and UPF1 (Figure 6B, α-SMG5), which requires serine phosphorylation in the SQ domain of UPF1. Coimmunoprecipitations indicated that the knockdown of MARVELD1 increased the binding of SMG5 by approximately 1.7 and 2.7-fold, and overexpression of MARVELD1 decreased the binding of SMG5 to approximately 70% of the level in the control cells (Figure 6B). Taken together, these results indicated that MARVELD1 likely blocks SMG5 complexes from being recruited to UPF1 by decreasing the serine phosphorylation of UPF1, which then inhibits nonsense-mediated RNA decay.

## Discussion

Here, we provide evidence that MARVELD1 represses nonsense-mediated RNA decay by blocking serine phosphorylation. Previously, we showed that the expression of MARVELD1 is remarkably downregulated in multiple tumors, especially in breast cancer and hepatocellular carcinoma [8,9]. However, the molecular function of MARVELD1 in normal tissues remains to be explored. Recently, the interaction and colocalization of MARVELD1 with CBP20 and importin β1 (data not shown) implied that MARVELD1 might also play a role in the same biological processes in which CBP20 and/or importin β1 participate, such as nuclear transport of protein or RNA, cap processing of pre-RNA, pre-mRNA splicing, and nonsense-mediated RNA decay (NMD) [1,11,12]. Because CBP20 and importin β1 are both involved in NMD, we first investigated the effect of MARVELD1 on NMD in this study. The transfection of NMD reporter plasmids indicated that MARVELD1 indeed inhibited NMD activity (Figure 1). The analysis of endogenous transcripts by real-time PCR showed that MARVELD1 also enhanced the stabilization of NMD target mRNAs but did not affect ORCL, which is not a substrate of NMD (Figure 2). Hereby, MARVELD1 likely regulates NMD.

MARVELD1 interacted with UPF1 and CBP20 but not SMG1 and CBP80 (Figure 3A and B) in the absence of RNA. This result implies that the interactions occur when CBP80 and SMG1 dissociate from CBP20 and UPF1. Considering that MARVELD1 promotes SMG1 release from UPF1 and does not affect the interaction between UPF1 and CBC (Figure 6, A), we presumed that MARVELD1 might bind to UPF1 before UPF1 interacts with CBP80, when SURF has released from CBC and is bound to the EJC, or MARVELD1 competes with UPF1 for binding to SMG1. Furthermore, SMG1 was immunoprecipitated by MARVELD1 in the presence of RNA but not in the absence of RNA (Figure 3A); we therefore infer that the association between SMG1 and MARVELD1 may not be a protein–protein interaction but an interaction requiring RNA as a bridge. That is, SMG1 and MARVELD1 bind to RNA independently either directly or indirectly, but they never exist in an identical protein complex at the same time and are likely to belong to distinct protein machineries that are both associated with RNA. We identified an interaction between MARVELD1 and the EJC component Y14 (Figure 3C, 3D and Figure S4), so it is likely that MARVELD1 competes with UPF1 for SMG1 binding when SURF is bound to the EJC. However, we do not know whether UPF1 or Y14 interacts with MARVELD1 first or whether MARVELD1 is a potential component of the EJC. With respect to the interaction between MARVELD1 and CBP20 (Figure 3A and B), we infer that it may occur when CBP80 and CBP20 have dissociated from each other because we did not observe the association between MARVELD1 and CBP80 by coimmunoprecipitation with/without crosslinking or by GST pulldown. Thus, although the interaction between MARVELD1 and CBP20 is a starting point to determine the potential function of MARVELD1 in NMD, more research is needed on the effect of the MARVELD1-CBP20 interaction on NMD. Future work will focus on supporting our speculations with additional experimental data.

Previously, the interaction of MARVELD1 and importin β1 was shown by liquid chromatography-tandem mass spectrometry analysis and coimmunoprecipitation [10], and it was suggested that MARVELD1 might participate in the biological function of importin β1. Additionally, importin β binds to CBP20 directly, influences cap binding and promotes the replacement of CBC by eIF4E [11,12]. Here, we showed that MARVELD1 interacts with UPF1 and enhances the binding of UPF1 to importin β1. Furthermore, MARVELD1 associates with CBP20 but not CBP80 (Figure 3A and B). Nevertheless, the mechanism through which MARVELD1 modulates the function of importin β1 during NMD remains to be clarified.

UPF1 primarily contains three important domains: the N-terminal domain is rich in cysteines and histidines (CH domain), the C-terminal region is rich in serine–glutamine clusters (SQ domain), and the helicase core is in the central region surrounded by these two domains. The helicase core is a conserved domain that unwinds double-stranded nucleic acids from the 5’- to 3’-end [25,26,27]. The CH domain provides a cis-acting inhibitory effect on the ATPase and unwinding activities of the helicase core region [25]. Recently, the molecular function of the SQ domain was elucidated; this region inhibits the ATP hydrolysis or duplex-unwinding ability of the UPF1 helicase core [28]. Meanwhile, several important serine–glutamine motifs in the SQ domain are phosphorylated by SMG1 in vivo in mammals, such as S1078, S1096 and S1116 [29,30], and phospho-S1096 is required for binding to SMG5-SMG7 and SMG5-PNRC2, which both result in the degradation of PTC-containing mRNAs [6,31]. In this study, we determined that MARVELD1 coimmunoprecipitates with the C-terminal region of UPF1 (Figure 5B) and that amino acids 34-69 of MARVELD1 are responsible for the interaction (Figure 4). Furthermore, we also showed that MARVELD1 disrupts the interaction between UPF1 and SMG1, reduces the serine phosphorylation of UPF1, and subsequently blocks the binding of SMG5 (Figure 6B). These data demonstrate that the most probable molecular mechanism through which MARVELD1 represses NMD activity is by disrupting the interaction of important NMD factors, such as SMG5, and UPF1 by blocking the phosphorylation of key serine sites, such as S1096.

Our findings indicate that MARVELD1 may be a novel regulator of the NMD pathway. However, it is unclear whether MARVELD1 stabilizes all NMD targets or only a subset. Although we analyzed the stabilization of only eight NMD target transcripts, the level of these mRNAs were substantially increased upon increased MARVELD1 expression, as were the NMD reporters pmCMV-G1 Ter and pmCMV-GPx1 Ter. Similar results were observed in different cell lines, including HeLa, HEK293, A549, H520 and HepG2. We therefore conclude that MARVELD1 indeed inhibits NMD. Nevertheless, MARVELD1 is a novel protein with uncharacterized molecular, and it is not yet known whether MARVELD1 always inhibits NMD or whether it functions only under certain circumstances, such as hyperphosphorylation or dephosphorylation. Future studies will uncover more detailed functions of MARVELD1 in NMD.

## Supporting Information

Table S1 Primers used to construct overexpression plasmids. (DOC)

Table S2 Primers used for real-time PCR. (DOC)

Figure S1 MARVELD1 enhances the stabilization of the NMD reporter GPx1 in four cell lines. (A) HepG2 cells stably overexpressing MARVELD1-V5 were transiently cotransfected with either pmCMV-Gl Norm or pmCMV-Gl Ter and phCMV-MUP. Expression analysis of MARVELD1-V5 was assessed by western blotting (right), and GAPDH was used as an internal control. (B) HEK293/MARVELD1 cells and HeLa and H520 cells both transiently depleted of MARVELD1 were transiently cotransfected with either pmCMV-GPx1 Norm or pmCMV-GPx1 Ter and phCMV-MUP. Real-time PCR was performed on cDNA 48 h after transfection. The levels of β-globin and GPx1 mRNA were both normalized to Mup mRNA, and the normalized level of Norm mRNA was defined as 1.0.
(TIF)

Figure S2 MARVELD1 does not affect steady-state translation. pGL4.73 [hRluc/SV40] vector was transfected into approximately 2×10^6^ cells either stably overexpressing MARVELD1(A) or transiently transfected with siRNAs against MARVELD1(B) for 48 h as well as control cells. The cells were harvested after another 48 h. Whole cell protein was extracted from half of the cells, and total RNA was collected from the other half. Renilla luciferase mRNA was subjected to RT-PCR using the primers listed in Table S2. Renilla luciferase activity was measured using whole cell protein according to the manufacturer’s instructions of the Dual-Luciferase Reporter Assay System (Promega). Luciferase activity was normalized to the level of luciferase mRNA to control for variations in transfection efficiencies.
(TIF)

Figure S3 The effects of RNase A displayed by RNA electrophoresis before or after RNase A treatment. Total RNA was purified from the extracts of approximately 1×10^7^ cells with or without RNase A treatment overnight at 4^°^C, and 2 μL of sample was used for electrophoresis.
(TIF)

Figure S4 Exogenous Flag-Y14 interacts with MARVELD1. (A) HeLa cells transiently transfected with pCMV-Flag-Y14 or the control plasmid pCMV-Flag were immunoprecipitated by anti-MARVELD1 or, as a control for nonspecific IP, rabbit (r) IgG in the presence of RNase A. (B) HEK293/MARVELD1-V5 cells or the control cells HEK293/Vector were transiently transfected with pCMV-Flag-Y14 and immunoprecipitated by anti-V5 in the presence of RNase A.
(TIF)

Figure S5 MARVELD1 decreases the phosphoserine level of endogenous UPF1. Lysates of HeLa cells transiently depleted of MARVELD1 and HEK293/MARVELD1 cells were immunoprecipitated by anti-UPF1.
(TIF)
