# Supplementary material for: MARVELD1 Inhibits Nonsense-Mediated RNA Decay by Repressing Serine Phosphorylation of UPF1
Source: PLoS One. 2013 Jun 27;8(6):e68291. doi: 10.1371/journal.pone.0068291 (PMC3694864; doi:10.1371/journal.pone.0068291)
Supplement: Table S1 — (DOC) [file pone.0068291.s001.doc]

**Table S1 Primers used to construct** overexpressing plasmids

| **Name** | **Sequence(5’-3’)** | **Reference** |
| --- | --- | --- |
| MARVELD1_F | GCGGATCCCATGCTCCCGCCGCCCCCGC | [9] |
| MARVELD1(1-33)_R | CGGAATTCTCA CACGCCCAGCGGGCTGCGCAG | This study |
| MARVELD1(1-69)_R | CGGAATTCTCACCAGAAGAGCACGGACACGAA | This study |
| MARVELD1(1-100)_R | CGGAATTCTCACGCCACGTTGACCATGAGCCAG | This study |
| MARVELD1(1-144)_R | CGGAATTCTCAGGCGGCCGCCAGGAAGGC | This study |
| MARVELD1(Δ34-69)_R1 | GAGGCCCAGGGTGAGCAGCACGCCCAGCGGGCTGCGC | This study |
| MARVELD1(Δ34-69)_F1 | GCGCAGCCCGCTGGGCGTGCTGCTCACCCTGGGCCTC | This study |
| MARVELD1_R | CCTCGAGTCACGCCACCTCCTGCTTGCCCTG | This study |
| UPF1_242_F | CGGAATTCCGCTGGTCAAGATCCCCTCCGAG | This study |
| UPF1_788_F | CGGAATTCCGGACCAGATTGGCATCATCACG | This study |
| UPF1_797_R | CCCTCGAGGTCACTCGTAGGGCGTGATGATGC | This study |
| UPF1_1118_R | CCGCTCGAGCGGTTAATACTGGGACAGCCCCGTC | This study |
