# Supplementary material for: MARVELD1 Inhibits Nonsense-Mediated RNA Decay by Repressing Serine Phosphorylation of UPF1
Source: PLoS One. 2013 Jun 27;8(6):e68291. doi: 10.1371/journal.pone.0068291 (PMC3694864; doi:10.1371/journal.pone.0068291)
Supplement: Table S2 — (DOC) [file pone.0068291.s002.doc]

Table S2 Primers used in real-time PCR

| **Name** | **Sequence(5’-3’)** | **Reference** |
| --- | --- | --- |
| β-globin-F | TGCACGTGGATCCTGAGAACTTCA | [13] |
| β-globin-R | ACCATTGTTCACAGGCAAGAGCAG | [13] |
| GPx1-F | CGGTTTCCCGTGCAATCAGTTCGG | [13] |
| GPx1-R | TCACCATTCACCTCGCACTTCTCA | [13] |
| Mup-F | CAGCTGATGGGGCTCTATG | [13] |
| Mup-R | TCCTAGTGAGAAGTCTCC | [13] |
| arhgef18-F | CCTACGCCAAGAAGCAAAAG | [18], [19] |
| arhgef18-R | CCCTGGAGTACACCTTCAGC | [18], [19] |
| ATF3-F | GCCATTGGAGAGCTGTCTTC | [18], [19] |
| ATF3-R | GGGCCATCTGGAACATAAGA | [18], [19] |
| DNAJb2-F | TGGCATCCTACTACGAGATCC | [18], [19] |
| DNAJb2-R | GTTTTTGTCTGGGTGCCACT | [18], [19] |
| ATF4-F | ATGTCCCCCTTCGACCA | This study |
| ATF4-R | CCATTTTCTCCAACATCCAATC | This study |
| Pim3-F | GCACCGCGACATTAAGGAC | This study |
| Pim3-R | TCCCCACACACCATATCGTAG | This study |
| Jag1-F | TTGCCCACTTTGAGTATCAGA | This study |
| Jag1-R | CAAGACCCATGCTTAGGACTG | This study |
| Frs2-F | TGTGGTGGAAGAGCCAGTTGT | This study |
| Frs2-R | CTGAAGGCAGGCGAGCAC | This study |
| Pisd-F | TCCCTGATGTCAGTGAACCCT | This study |
| Pisd-R | TGGTGTGCGTCACGAAGC | This study |
| ORCL-F | GGCAGCAGATGAAATCTGAA | [18], [19] |
| ORCL-R | TCCAGAATGTGATTTTTGCAG | [18], [19] |
| GAPDH-F | AACAGCCTCAAGATCATCAGC | [9] |
| GAPDH-R | GGATGATGTTCTGGAGAGCC | [9] |
| Renilla-F | ATGGCTTCCAAGGTGTACGA | This study |
| Renilla-R | GATCCAGGAGGCGATATGAG | This study |
