# Supplementary figures and images for: MARVELD1 Inhibits Nonsense-Mediated RNA Decay by Repressing Serine Phosphorylation of UPF1
Source: PLoS One. 2013 Jun 27;8(6):e68291. doi: 10.1371/journal.pone.0068291 (PMC3694864; doi:10.1371/journal.pone.0068291)

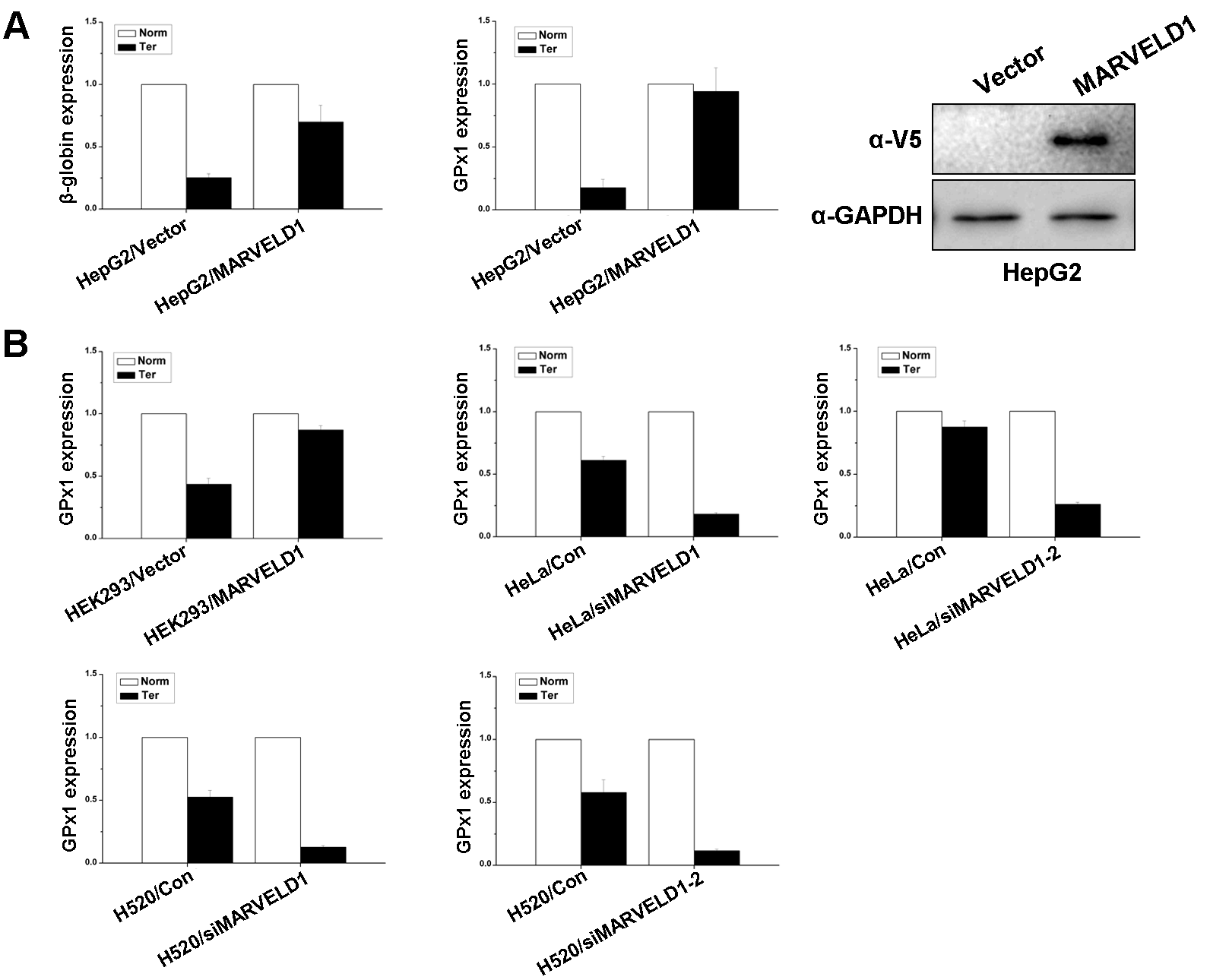

Supplement: Figure S1 — (A) HepG2 cells stably overexpressing MARVELD1-V5 were transiently cotransfected with either pmCMV-Gl Norm or pmCMV-Gl Ter and phCMV-MUP. Expression analysis of MARVELD1-V5 was assessed by western blotting (right), and GAPDH was used as an internal control. (B) HEK293/MARVELD1 cells and HeLa and H520 cells both transiently depleted of MARVELD1 were transiently cotransfected with either pmCMV-GPx1 Norm or pmCMV-GPx1 Ter and phCMV-MUP. Real-time PCR was performed on cDNA 48 h after transfection. The levels of β-globin and GPx1 mRNA were both normalized to Mup mRNA, and the normalized level of Norm mRNA was defined as 1.0. (TIF) [file pone.0068291.s003.tif]

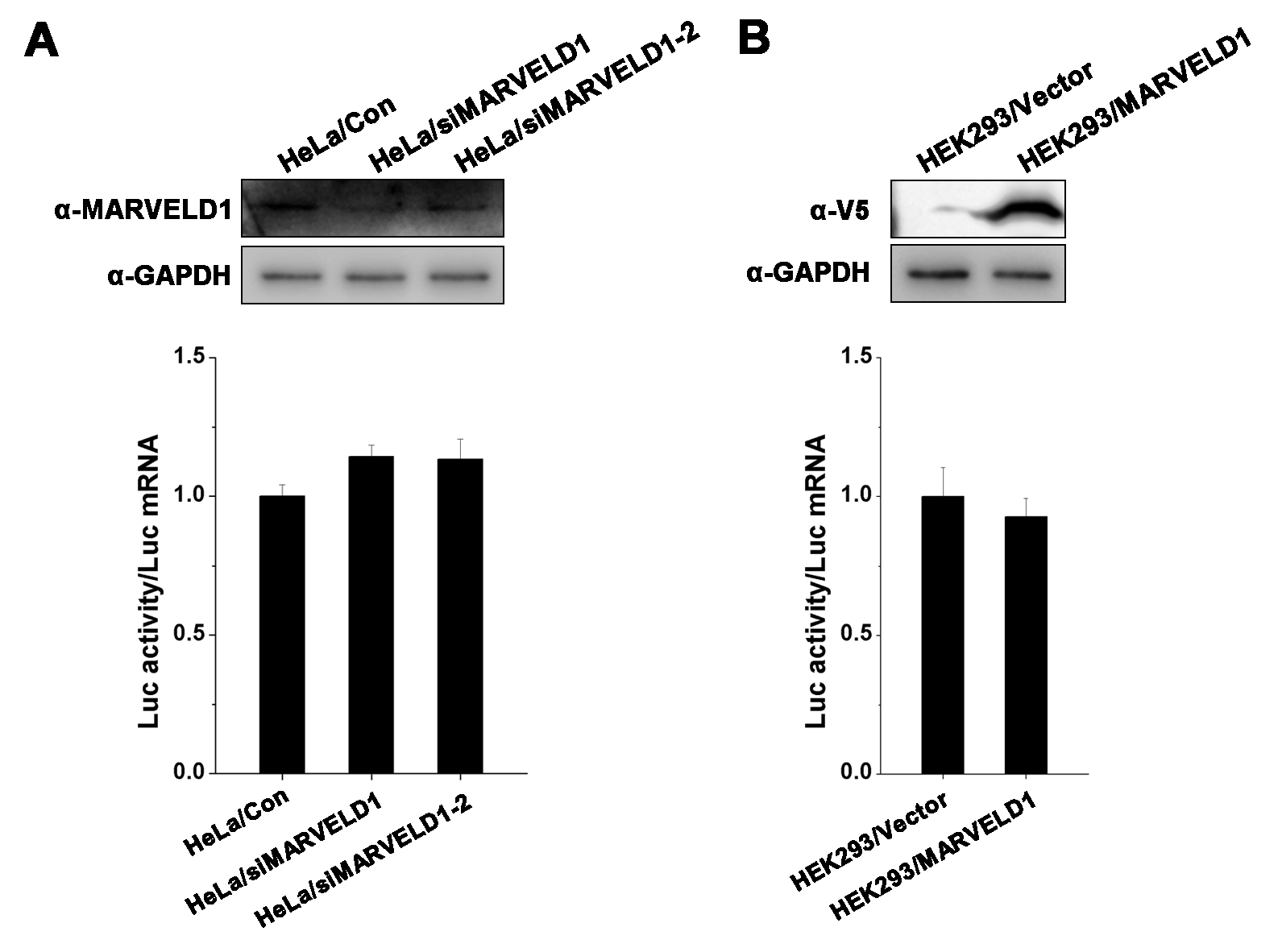

Supplement: Figure S2 — pGL4.73 [hRluc/SV40] vector was transfected into approximately 2×10^6^ cells either stably overexpressing MARVELD1(A) or transiently transfected with siRNAs against MARVELD1(B) for 48 h as well as control cells. The cells were harvested after another 48 h. Whole cell protein was extracted from half of the cells, and total RNA was collected from the other half. Renilla luciferase mRNA was subjected to RT-PCR using the primers listed in Table S2. Renilla luciferase activity was measured using whole cell protein according to the manufacturer’s instructions of the Dual-Luciferase Reporter Assay System (Promega). Luciferase activity was normalized to the level of luciferase mRNA to control for variations in transfection efficiencies. (TIF) [file pone.0068291.s004.tif]

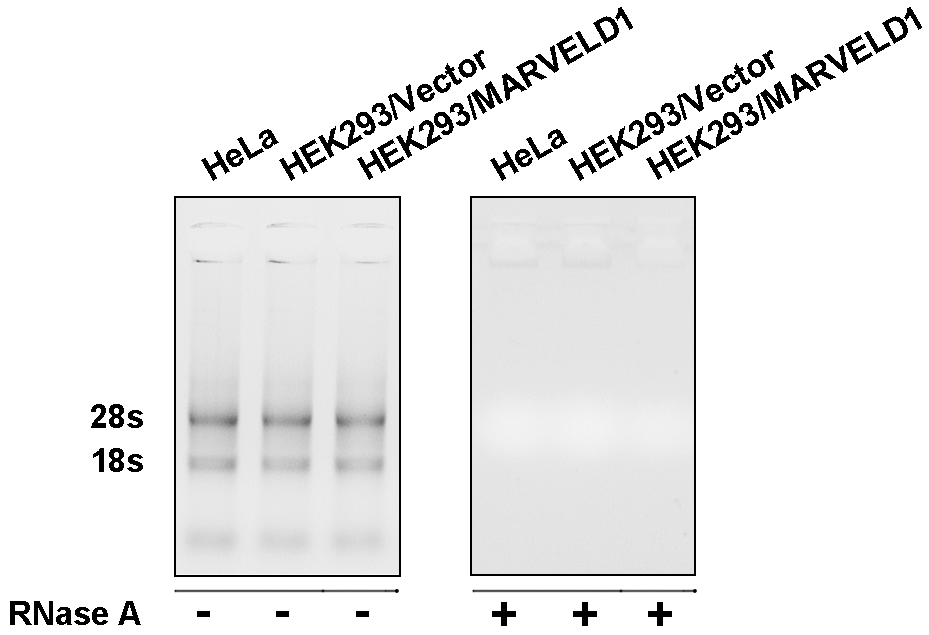

Supplement: Figure S3 — Total RNA was purified from the extracts of approximately 1×10^7^ cells with or without RNase A treatment overnight at 4°C, and 2 μL of sample was used for electrophoresis. (TIF) [file pone.0068291.s005.tif]

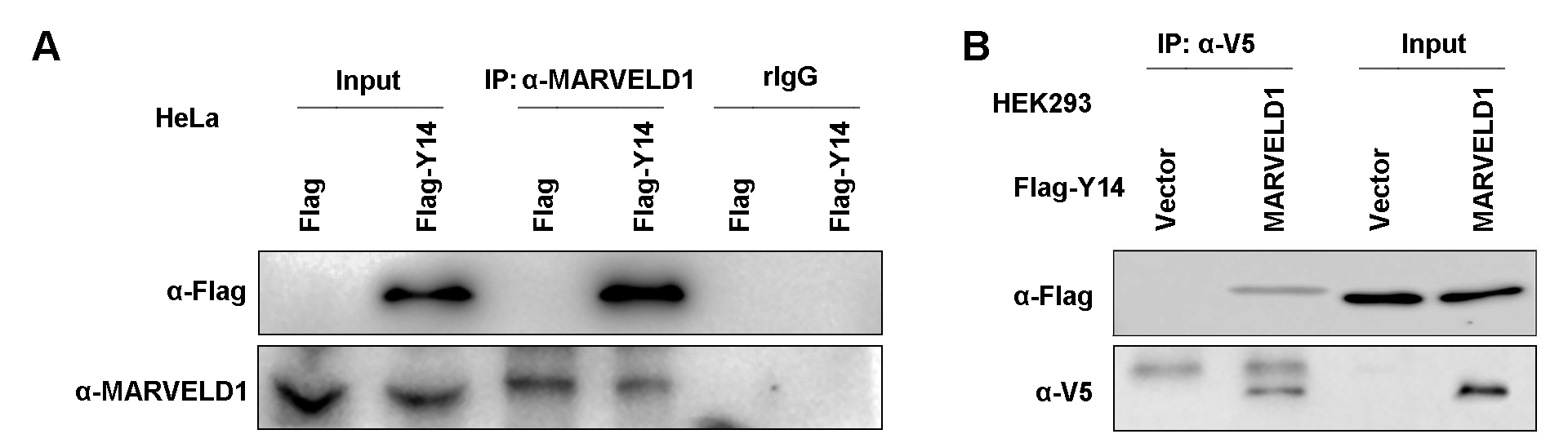

Supplement: Figure S4 — (A) HeLa cells transiently transfected with pCMV-Flag-Y14 or the control plasmid pCMV-Flag were immunoprecipitated by anti-MARVELD1 or, as a control for nonspecific IP, rabbit (r) IgG in the presence of RNase A. (B) HEK293/MARVELD1-V5 cells or the control cells HEK293/Vector were transiently transfected with pCMV-Flag-Y14 and immunoprecipitated by anti-V5 in the presence of RNase A. (TIF) [file pone.0068291.s006.tif]

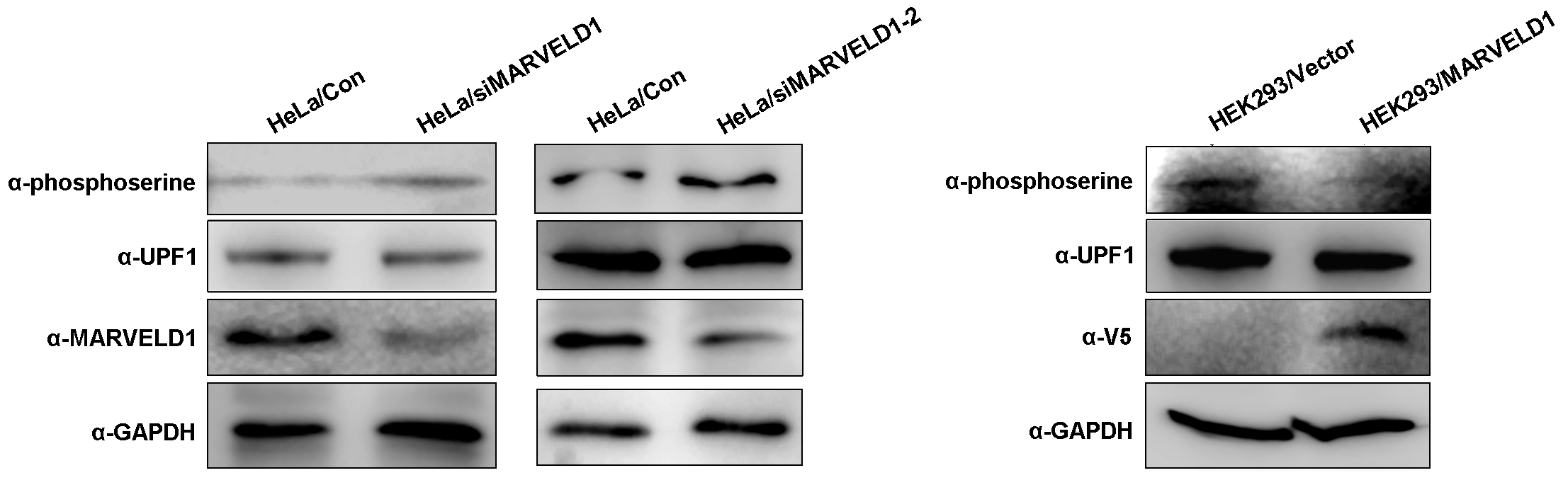

Supplement: Figure S5 — Lysates of HeLa cells transiently depleted of MARVELD1 and HEK293/MARVELD1 cells were immunoprecipitated by anti-UPF1. (TIF) [file pone.0068291.s007.tif]
